# Investigation of the optimal method of oxygen administration with simultaneous use of a surgical mask: a randomized control study

**DOI:** 10.1007/s00540-021-02998-6

**Published:** 2021-09-07

**Authors:** Yusuke Matsui, Tomonori Takazawa, Akihito Takemae, Yukie Murooka, Masafumi Kanamoto, Shigeru Saito

**Affiliations:** 1 Department of Anesthesiology, Gunma University Graduate School of Medicine, Maebashi, Japan; 2 Intensive Care Unit, Gunma University Hospital, 3-39-15 Showa-machi, Maebashi, Gunma 371-8511 Japan

**Keywords:** Surgical mask, Oxygen administration, Oxygen reserve index, COVID-19

## Abstract

**Purpose:**

From the perspective of infection prevention during the Coronavirus disease 2019 (COVID-19) pandemic, a recommendation was made to use surgical masks after extubation in patients in the operating room. For compliance with this recommendation, anesthesiologists need to administer oxygen to the patient with an oxygen mask over the surgical mask. However, no studies have investigated whether this method allows good maintenance of oxygenation in patients. This study aimed to investigate which method of oxygen administration lends itself best to use with a surgical mask in terms of oxygenation.

**Method:**

We administered oxygen to the study subjects using all the following three methods in random order: an oxygen mask over or under a surgical mask and a nasal cannula under the surgical mask. Oxygenation was assessed using the oxygen reserve index (ORi) and end-tidal oxygen concentration (EtO_2_).

**Result:**

This study included 24 healthy volunteers. ORi values with administration of oxygen were higher in the order of a nasal cannula under the surgical mask, an oxygen mask under the surgical mask, and an oxygen mask over the surgical mask, with median values of 0.50, 0.48, and 0.43, respectively, and statistically significant differences between all groups (*P* < 0.001). EtO_2_ values were in the same order as ORi, with median values of 33.0%, 31.0%, and 25%, respectively, and statistically significant differences between all groups (*P* < 0.001).

**Conclusion:**

Wearing a surgical mask over the nasal cannula during oxygen administration is beneficial for oxygenation and might help prevent aerosol dispersal.

**Supplementary Information:**

The online version contains supplementary material available at 10.1007/s00540-021-02998-6.

## Introduction

The global Coronavirus disease 2019 (COVID-19) pandemic has resulted in an increase in mask usage for infection prevention according to global health recommendations [1–3]. Accordingly, from the perspective of preventing infections in healthcare workers and other patients, patients receiving oxygen administration in the operating room are not exempt from this recommendation. The Japanese Society of Anesthesiologists recommended administering oxygen using an oxygen mask worn over a surgical mask in all patients after extubation [4]. When combining oxygen administration with use of a surgical mask, both administration of oxygen from over or under the surgical mask can be considered. A previous study reported no significant difference in inspiratory oxygen fraction when oxygen was administered over or under the surgical mask [5]. However, another report showed different results: oxygen administration with the oxygen mask over the surgical mask resulted in lower inhaled oxygen levels than with its administration under the surgical mask [6].

In daily practice, we experienced cases in which oxygenation worsened when oxygen was administered over the surgical mask. In such cases, administering oxygen using a nasal cannula under the surgical mask often improved patient oxygenation. These varying results inspired us to compare the administration of oxygen to COVID-19 patients using an oxygen mask over the surgical mask and a nasal cannula under the surgical mask. The results showed that arterial partial pressure of oxygen (PaO_2_) increased when oxygen was administered by a nasal cannula under the surgical mask [7]. However, our report was insufficient to develop a clear strategy for the optimal oxygen administration method since the report was based on data from a single patient. Furthermore, it was difficult to compare the effectiveness of oxygen administration over and under the surgical mask, because the administration methods involved different devices: an oxygen mask and a nasal cannula.

This study aimed to investigate the effect of simultaneous use of a surgical mask during oxygen administration on patient oxygenation, and to determine whether an oxygen mask or nasal cannula is more advantageous when oxygen is administered under the surgical mask. To achieve this, we administered oxygen to healthy volunteers in the following three ways: via an oxygen mask used over or under the surgical mask and via nasal cannula under the surgical mask. Oxygenation was assessed as the oxygen reserve index (ORi) [8, 9] and end-tidal oxygen concentration (EtO_2_) [7, 10–12].

## Methods

### Ethics approval

This randomized, single-blind, cross-over study conformed to the standards of the Declaration of Helsinki and was approved by the ethics committee of Gunma University Hospital (Trial No. IRB2021-001). The study was registered with the University Hospital Medical Information Network Clinical Trials Registry (UMIN000044274). The subjects participated on a voluntary basis, and written informed consent was obtained from each participant.

### Subjects

Inclusion criteria were: (1) age between 20 and 60 years, and (2) legally competent to consent. Exclusion criteria were (1) history of respiratory illness, (2) smokers, (3) percutaneous arterial oxygen saturation of less than 92% before the measurement, and (4) exhaled carbon dioxide partial pressure of less than 28 mmHg or more than 45 mmHg.

Based on our pilot study, the required sample size was 23 subjects with power analysis using *α* = 0.05 and *β* = 0.8. Assuming a 5% dropout rate, the plan was to enroll 24 participants.

### Oxygen administration method

Oxygen was administered at a flow rate of 4 L/min with an oxygen mask (Japan Medicalnext Co. Ltd., Osaka, Japan, catalog No. 001421) and oxygen cannula (Japan Medicalnext Co. Ltd., catalog No. 001594). Pro-Lane^®^ Level-1 (Medicom Japan Inc. Ltd., Kobe, Japan), which is the surgical mask we use in our daily practice and meets level 1 of the American standards for medical masks, the American Society for Testing and Materials (ASTM), was used as the surgical mask [13]. This mask has a particle filtration efficacy (PFE), an indicator of the ability to collect particles with a diameter of 0.1 μm, of higher than 98%, indicating that it has sufficient performance to collect small particles.

**Fig. 1:**
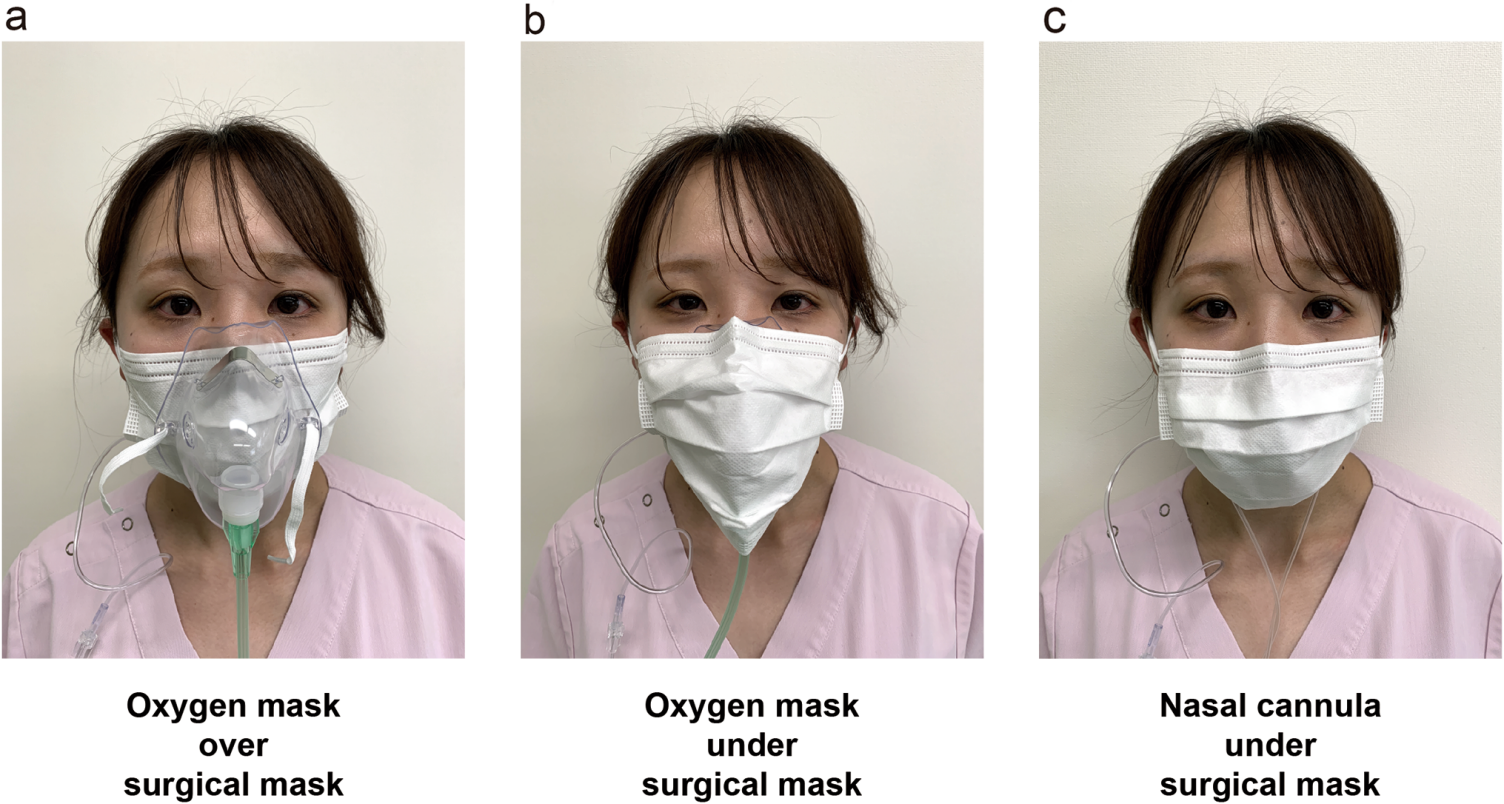
The three different oxygen administration methods tested. Image showing wearing an oxygen mask over (**a**) and under (**b**) the surgical mask and wearing a nasal cannula under the surgical mask (**c**). The subject held a gas sampling tube in her mouth

**Fig. 2:**
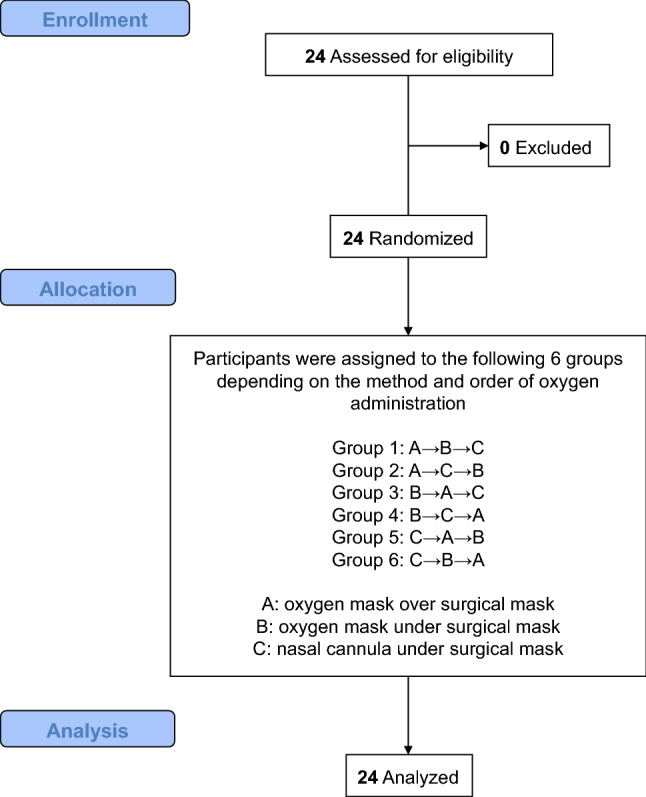
Subject recruitment, randomization and analysis

Oxygen was administered by the following three methods (Fig. 1): via an oxygen mask over the surgical mask, with an oxygen mask under the surgical mask, and via a nasal cannula under the surgical mask. Participants received oxygen once by each method, and the order of administration was randomly selected from one of six ways using the envelope method (Fig. 2). In the envelope method, a piece of paper with the order of oxygen administration is placed inside the envelope, and oxygen is administered in the order indicated on the paper. This randomization allowed us to eliminate the potential risk of systematic error that might arise from fixing the order of oxygen administration methods. For single blinding, the oxygen administration method was made invisible to the recorder using a partition.

### Measurement method and endpoints

The primary endpoint of this study was the ORi and the secondary endpoint was EtO_2_. The ORi has been shown to correlate with PaO_2_ and is an index that can be used to estimate PaO_2_ even in the moderate hyperoxic range (PaO_2_ of approximately 100–200 mmHg) [8, 9]. We measured ORi with a pulse co-oximeter sensor placed on the patient’s index finger and connected to a Radical-7^®^ pulse oximeter (Mashimo Corp., Irvine, CA, USA).

During the study, the subjects were instructed to hold a gas sampling tube in their mouth. Due to technical difficulties, we compared oxygen levels in exhaled air (EtO_2_) rather than inhaled air. Assuming that oxygen consumption during the measurements is constant, the difference in EtO_2_ can be considered to reflect the difference in inhaled oxygen concentration [7]. Therefore, EtO_2_ has previously been used as an index of preoxygenation before general anesthesia [10–12]. A multi-gas analysis unit (GF-320R, Nihon Kohden Corporation, Tokyo, Japan) was used for exhaled gas analysis. The same person was in charge of the recordings in all cases. ORi and EtO_2_ were measured at the following three points: at least 90 s after the start of oxygen administration and when the recorder judged that the oxygen concentration had stabilized (T1), 10 s after T1 (T2), and 20 s after T1 (T3). All values from T1 to T3 were used to analyze each individual oxygen administration method to minimize the risk of accidentally adopting only outliers.

### Statistical analyses

Differences in ORi and EtO_2_ between each oxygen administration method were analyzed using Friedman repeated measures analysis of variance on ranks with a post hoc Tukey test. Sigmaplot 14.5 (Systat Software Inc., San Jose, CA) was used for the analysis. Differences were considered significant at a *P* value of < 0.05.

## Results

A total of 24 healthy volunteers participated in the study, and not a single person dropped out of the study (Fig. 2). Subject background characteristics are shown in Supplemental Table 1. Briefly, mean values of age, height, weight, and body mass index were 30.2 years, 162.4 cm, 55.7 kg, and 21.1 kg/m^2^, respectively.

**Fig. 3:**
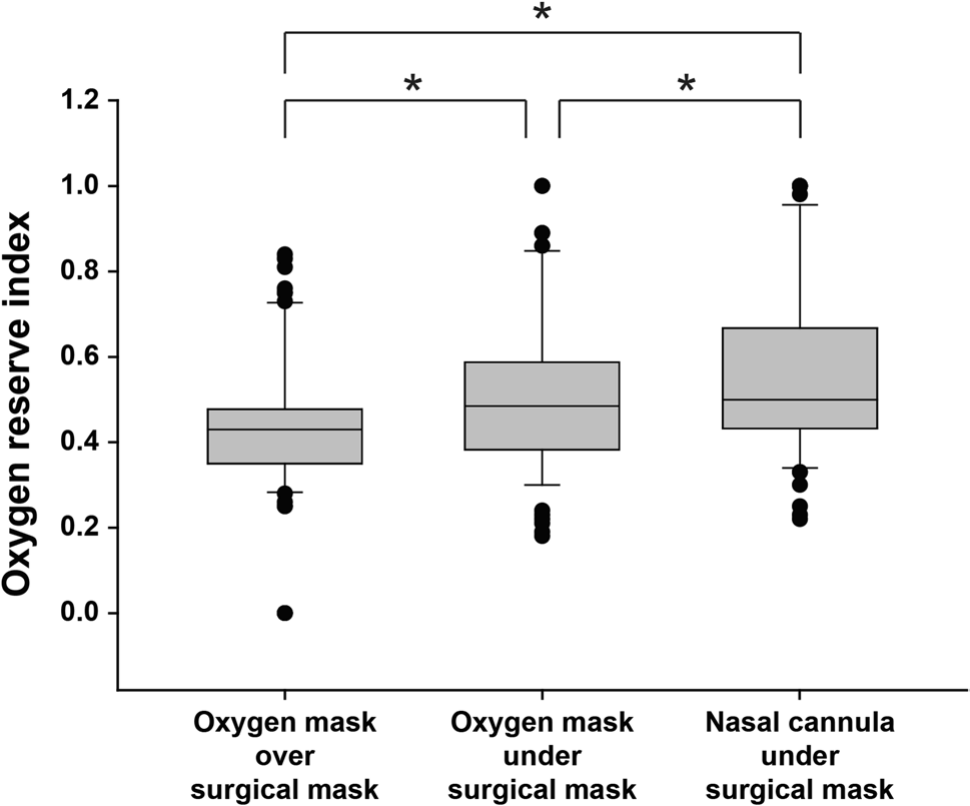
Comparison of oxygen reserve index between the three oxygen administration methods. The ends of the box define the 25th and 75th percentiles, with the horizontal line in the middle showing the median, and the error bars defining the 10th and 90th percentiles. The dots indicate outliers. *Friedman repeated measures analysis of variance on ranks with the post hoc Tukey test, *P* < 0.001

First, we analyzed ORi with the different oxygen administration methods. With oxygen administration using an oxygen mask, ORi was greater with oxygen delivery under the surgical mask rather than over the mask (Fig. 3, median value 0.48 vs. 0.43, *P* < 0.001). When comparing the nasal cannula and oxygen mask applied under the surgical mask, ORi was greater when oxygen was administered via the nasal cannula (Fig. 3, median value 0.50 vs. 0.48, *P* < 0.001).

**Fig. 4:**
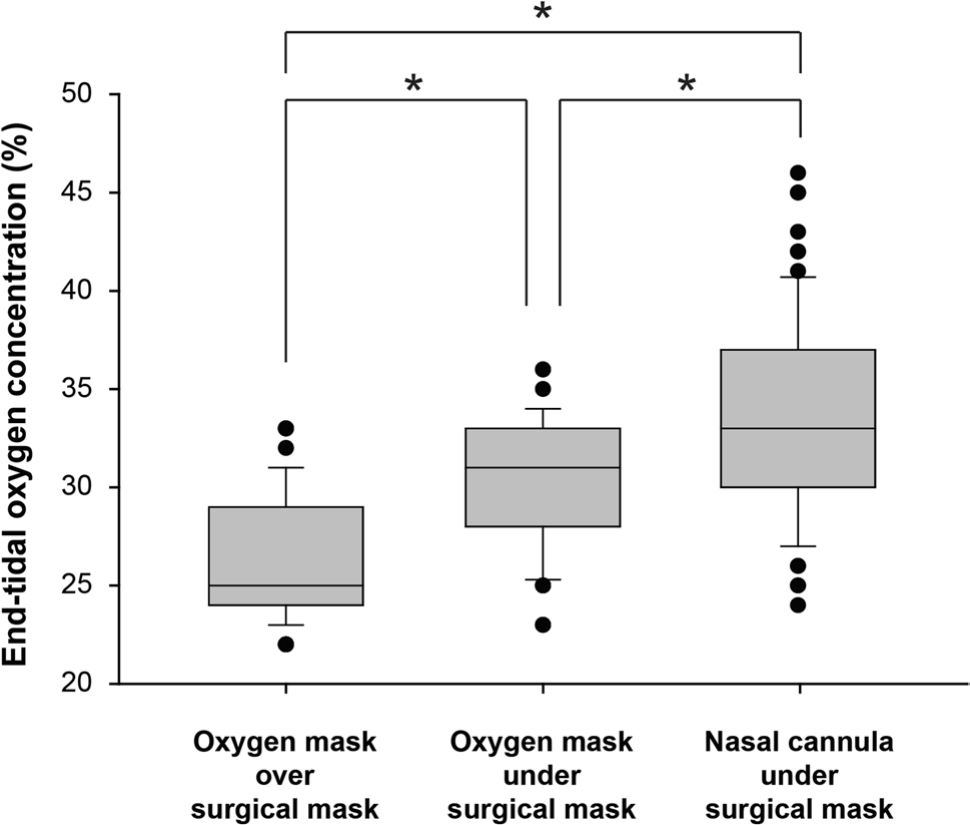
Comparison of end-tidal oxygen concentration between the three oxygen administration methods. The ends of the box define the 25th and 75th percentiles, with the horizontal line in the middle showing the median, and the error bars defining the 10th and 90th percentiles. The dots indicate outliers. *Friedman repeated measures analysis of variance on ranks with the post hoc Tukey test, *P* < 0.001

In the analysis of EtO_2_, with oxygen administration via the oxygen mask, EtO_2_ was greater with oxygen delivery under the surgical mask than over the mask (Fig. 4, median value 31.0% vs. 25.0%, *P* < 0.001). When comparing the nasal cannula and oxygen mask applied under the surgical mask, EtO_2_ was greater when oxygen was administered by the nasal cannula (Fig. 4, median value 33.0% vs. 31.0%, *P* < 0.001).

## Discussion

This study showed that when 4 L/min of oxygen was administered using an oxygen mask, oxygen administration over the surgical mask resulted in worse oxygenation than when the surgical mask was placed over the oxygen mask. Furthermore, a comparison of oxygen masks and nasal cannulas revealed that a nasal cannula applied under the surgical mask provided better oxygenation than oxygen delivery via an oxygen mask worn under the surgical mask. This is the first randomized controlled trial to compare oxygenation with oxygen administered using an oxygen mask and nasal cannula worn under the surgical mask.

There have been reports of how oxygenation is affected by the combined use of a surgical mask with an oxygen mask [5, 6]. However, the number of subjects included in each report was small, and the results of those reports were inconsistent. Therefore, for precise examination, statistical errors were minimized by including a sufficient number of subjects in this study. Furthermore, we controlled for information bias by ensuring that the person recording the data was blinded to the oxygen administration method used. With these measures, we believe that we have credible data on the position of the surgical mask that is advantageous for oxygenation when oxygen is administered via an oxygen mask. Although the mechanism by which oxygenation worsens when oxygen is administered over a surgical mask was not the focus of this research, this could be explained by dilution of the administered gas mixture by room air [14].

In this study, we also compared oxygen masks and nasal cannulas. A previous study comparing oxygen administration using oxygen masks and nasal cannulas in postoperative patients, although without use of a surgical mask, reported that SpO_2_ was higher with nasal cannulas [15, 16]. The results of this previous study support our findings. However, since it is unclear why nasal cannulas are advantageous for oxygenation, further studies are needed to elucidate the mechanism.

Although previous studies have all used a single index to assess oxygenation, we used two indices, ORi and EtO_2_, to improve the accuracy of the evaluation. ORi has been increasingly used in recent years, because it allows non-invasive assessment of oxygenation at a range that cannot be assessed by SpO_2_ [17]. In this study, we decided to use ORi to assess oxygenation because all the subjects were healthy people, and SpO_2_ was expected to exceed 100% after 4 L/min of oxygen administration in most cases. In fact, when oxygen was administered via the nasal cannula, which had the greatest median ORi, only 25% of the subjects had an ORi value of 0.67 or more (Fig. 3). The predicted PaO_2_ value for an ORi of 0.67 is approximately 210 mmHg [9]. Since the correlation between ORi and PaO_2_ is fair when PaO_2_ is less than 240 mmHg [9], it was reasonable to use ORi as an index of oxygenation in this study. Of course, while PaO_2_ is the best way to assess oxygenation, it would have been ethically inappropriate to perform a highly invasive experiment since the present study was conducted on healthy volunteers.

Endotracheal extubation and subsequent oxygen administration are considered aerosol generating procedures in healthcare settings [18]. The reason for recommending surgical masks for patients after extubation is to control the spread of aerosols generated by the patient and prevent secondary infections among healthcare workers [4]. A recent study in silico suggested that wearing a surgical mask over the nasal cannula during oxygen therapy significantly reduced aerosol dispersal [19]. Although aerosol experiments were not performed in the present study, wearing a surgical mask over the nasal cannula would impair aerosol dispersal.

A limitation of this study is that the effect of oxygen administration was only examined at an oxygen flow rate of 4 L/min. We chose one oxygen flow rate to simplify the experimental protocol. Another reason was that we predicted that if the oxygen flow rate was higher than 4 L/min, the PaO_2_ would be higher than 240 mmHg, and some subjects would fall outside the optimal measurement range of ORi [9]. Although different flow rates of administered oxygen might change the results, there was no significant difference in a similar study when the oxygen flow rate was varied from 5 and 7 to 10 L/min [6].

Differences in oxygenation between the three oxygen administration methods might not be a problem in healthy individuals. Indeed, in the case of subjects who participated in this study, the median PaO_2_ estimated using ORi was 160 mmHg when oxygen was administered with an oxygen mask over the surgical mask [9]. On the other hand, the median predicted PaO_2_ when oxygen is administered via nasal cannula under the surgical mask is 175 mmHg [9]. However, our results cannot be directly extrapolated to patients immediately after anesthesia and extubation, since such patients might not have fully recovered from the effects of anesthesia and might be hypoventilated, and some patients might have poor oxygenation capacity even before surgery. In such cases, oxygen administration by a nasal cannula applied under the surgical mask would be desirable. Nevertheless, it is necessary to verify whether the results of this study also apply to patients after general anesthesia.

In conclusion, wearing a surgical mask over the nasal cannula during oxygen administration is beneficial for oxygenation and might help prevent aerosol dispersal.

## Supplementary Information

Below is the link to the electronic supplementary material.

Supplementary file1 (DOCX 14 kb)
