# Supplementary material for: Investigation of the optimal method of oxygen administration with simultaneous use of a surgical mask: a randomized control study
Source: J Anesth. 2021 Sep 7;36(1):26–31. doi: 10.1007/s00540-021-02998-6 (PMC8422829; doi:10.1007/s00540-021-02998-6)
Supplement: Supplementary file 1 — Supplementary file1 (DOCX 14 kb) [file 540_2021_2998_MOESM1_ESM.docx]

Supplemental Table 1 Baseline characteristics of the study subjects

|  | Total (n = 24) | Male (n = 8) | Female (n = 16) |
| --- | --- | --- | --- |
| Age (yr) | 30.2 ± 6.0 | 29.0 ± 3.8 | 30.8 ± 6.8 |
| Height (cm) | 162.4 ± 12.8 | 169.4 ± 7.2 | 158.9 ± 8.4 |
| Weight (kg) | 55.7 ± 14.9 | 62.8 ± 11.6 | 52.2 ± 10.9 |
| Body mass index (kg/m^2^) | 21.1 ± 4.0 | 21.9 ± 4.1 | 20.7 ± 3.8 |
